# The impact of reimbursement negotiations on cost and availability of new pharmaceuticals: evidence from an online experiment

**DOI:** 10.1186/s13561-020-00267-y

**Published:** 2020-05-21

**Authors:** Dominik J. Wettstein, Stefan Boes

**Affiliations:** Department of Health Sciences and Medicine, University of Lucerne, Frohburgstrasse 3, P.O. Box 4466, CH-6002 Lucerne, Switzerland

**Keywords:** Reimbursement, Negotiation, Willingness to pay, Willingness to accept, Social preferences, Health insurance, QALY, Value-based pricing, Health technology assessment

## Abstract

**Background:**

The necessity to measure and reward “value for money” of new pharmaceuticals has become central in health policy debates, as much as the requirement to assess the “willingness to pay” for an additional, quality-adjusted life year (QALY). There is a clear need to understand the capacity of “value-based” pricing policies to impact societal goals, like timely access to new treatments, sustainable health budgets, or incentivizing research to improve patient outcomes. Not only the pricing mechanics, but also the process of value assessment and price negotiation are subject to reform demands. This study assesses the impact of a negotiation situation for life-extending pharmaceuticals on societal outcomes. Of interest were general effects of the bargaining behaviour, as well as differences caused by the assigned role and the magnitude of prices.

**Methods:**

We ran an online experiment (*n* = 404) on Amazon Mechanical Turk (MTurk). Participants were randomly assigned into four treatment groups for a reimbursement negotiation between two roles (health minister, pharma representative) in two price framings. Payoff to players consisted of a fixed salary and a potential bonus, depending on their preferences, their price offer and the counter offer of a randomly paired negotiation partner. Success had real social consequences on other MTurk users (premium payers, investors) and via donations to a patient association.

**Results:**

Margins between reservation prices and price offers increased throughout the game. Yet, 47% of players reduced at least once and 15% always their bonus probability to zero in favour of an agreement. 61% of simulated negotiation pairs could have reached an agreement, based on their preferences. 63% of these were successful, leaving 61% of patients with no access to the new treatment. The group with “real world” prices had lower prices and less agreements than the unconverted payoff group. The successful markets redistributed 20% of total assets from premium payers to investors over five innovation cycles.

**Conclusions:**

The negotiation situation for pharmaceutical reimbursement has notable impact on societal outcomes. Further research should evaluate policies that align preferences and increase negotiation success.

## Introduction

Health authorities and health care payers in OECD countries face an increasing number of new medicines offered at high prices [1, 2]. Especially in oncology, the pipelines of pharmaceutical companies have grown in the past decade by almost 80%, reaching a “historic high level” of 849 molecules in late-stage [3, 4]. This brings an increasing challenge for regulators and payers to secure access for patients to new, lifesaving treatments while controlling expanding costs. The necessity to measure and reward “value for money” has become central in health policy debates, as much as the requisite for appropriate methods to assess the “willingness to pay” (WTP) for an additional, quality-adjusted life year [1, 5–10]. Not only the policy outcomes, but also the process of value assessment and price negotiation is subject to reform demands [1, 11–15]. While European countries seek to collaborate more in reimbursement decisions [1, 2, 10, 12], pharmaceutical industry expresses concerns about “significant variance in access to new medicines across Europe” [16, 17].

## Background

### Behavioural perspective

This study aims to enrich the reform debate on pharmaceutical pricing from a behavioural perspective. In the past 30 years behavioural economic studies have delivered evidence on how individuals deviate in different ways from the assumption of neoclassical models [18–21]. Of interest are for example deviations from standard preferences and standard decision making due loss aversion, framing effects, anchoring effects and concerns for others etc. [20, 22]. In recent years the heuristics and implications have been incorporated into public policy analysis and even policy implementation in different countries, including health care [21, 23–27]. Just recently it has been argued, that “several behavioural economic-related phenomena may affect price negotiations […] between pharmaceutical ‘buyers’ and ‘sellers’” [14]. However, the experimental research on pharmaceutical pricing negotiations is still scarce to non-existing [15, 28]. The main reason for this is of course that the respective interactions take place in a much more complex setting than in other markets [15].

Our experimental design [15] integrates three established fields of research around willingness to pay (WTP) and willing to accept (WTA): assessment of QALY preferences (WTP for health), assessments of exchange asymmetries (WTP vs. WTA) and assessment of social preferences (WTA or WTP reflecting distributional effects). The setting allows us to measure underlying preferences, incentivized offer statements, as well as the societal effects of reimbursement negotiations for new life-extending pharmaceuticals. For more background on the three fields of research see our previous study [15].

### Objective

Aim of this study is to assess the impact of the negotiation situation for life-extending pharmaceuticals on societal outcomes. Of interest are general effects of the bargaining behaviour, as well as differences caused by the assigned role in a decision situation with incremental changes of the patient outcome. The study was not designed to infer generalizable WTP per QALY values in oncology but to assess selected influencing factors of the negotiation situation. Since there is a clear need for empirical evidence on the effectiveness of pharmaceutical pricing policies, particularly for value-based pricing [28], we deem our experiment a starting point for further investigations on policy interventions that aim to improve negotiation outcomes. Our previous systematic literature review revealed no published results from any experiments on pharmaceutical pricing negotiations [28]. A discussion on external validity for current policy debates follows at the end of the paper.

### Preferences in reimbursement negotiations

This study builds on a previous study where we reported the effects of the negotiation situation on stated preferences [15]. Findings showed that the framed price magnitude of current oncology treatments has an impact on stated preferences for incremental survival. Players assigned to the role of the regulator (“health minister”) stated lower prices in the fictive “real world” prices (100 k$) group compared to their colleagues in the “real payoff” prices (1$) group. For players in the seller role (pharmaceutical representative), the effect was not significant. We found no systematic valuation gaps (“reluctance to trade” with WTP < TWA) between the two negotiating roles. In contrast, regulators in the 1$ group showed a tendency for lower reservation prices than their counterparts. However, the assigned responsibility had an impact on the reported relevance of the stakeholders affected (the patient, the premium payers, the investors, the opponent and the own role) for the decision. Regulators rated the patient higher and more often as most important while the own role was rated higher in sellers. Finally, we found no evidence for any interest in effectiveness or efficiency by participants. Mean incremental cost-effectiveness ratio (ICER) versus standard of care (SoC) was not stable and higher in all rounds compared to the initial ICER of the SoC versus no treatment.

The previous study focused on stated preferences in the negotiation. This study will complete the setting with incentivized bargaining offers and a matching of the offer statements.

## Methods

### Design

In the following we provide a summary of our experimental research design, described in more details in our previous study [15].

We ran an online experiment (*n* = 404) on the Amazon Mechanical Turk (MTurk) platform. Convincing evidence exists regarding the reliability of MTurk results compared to laboratory and field experiments, also specifically for assessments of social preferences [29–36]. Participants were randomly assigned into four treatment groups of equal size (Table 1). Group one and three played the games as health ministers (regulators), while group two and four played as representatives of a pharmaceutical company (sellers). The groups were further separated into two different price magnitude framings (the 100 k$ group with fictive “real world” prices of oncology treatments and the 1$ group). Final payoff functions were equal for both groups, but the 100 k$ played with a conversion of the payoffs of 100,000$ = 1USD during the game (we use the prices of this group in the following explanations). To implement social effects, participants were given full information on how their decision would have an impact in real life on others: on other MTurk users for premium payers or investors and via donations to a patient association as a proxy for patient benefits.

**Table 1:** Number of subjects randomly assigned to treatment groups

Game	Outcome	Groups (n)			
		Group 1	Group 2	Group 3	Group 4
Game currency to real payoff		100,000 $ = 1 US$		1 $ = 1 US$	
Role		Regulator	Seller	Regulator	Seller
Round 1 to 5 (1st game)	Reser-vation price (x)	WTP (97)	WTA (101)	WTP (105)	WTA (101)
Round 1 to 5 (2nd game)	Price offer (y)	Offer (97)	Offer (101)	Offer (105)	Offer (101)

*WTP* Willingness to pay, *WTA* Willingness to accept

Participants played the same five rounds in two consecutive games. All relevant information about the consequences of the negotiation were provided before the first game. Participants did not know which game would be relevant for final payoffs, nor that the game would be repeated after five rounds to prevent strategic behaviour

The reimbursement negotiation plays in a hypothetical country and involves seven stakeholders:

A patient, suffering from a deadly blood cancer, already under treatment with a reimbursed pharmaceutical (current standard of care, SoC) with a known benefit. A regulator responsible for regulating prices of new pharmaceuticals, approved for payment by public health insurance. A seller, employed by an international pharmaceutical company, commissioned to negotiate a price for a new product to treat the patient. Two premium payers who fund public health insurance. Their accumulated premiums are the solitary funding source for the patient’s treatment (no out-of-pocket payment possible). Two investors of the pharmaceutical company who fund the new therapy. They expect a return on their investment, which compensates them for the risk of investment.

In each round of the experiment, the seller offers a given new treatment at a proposed price, while the regulator (simultaneously) proposes an appropriate price for the same treatment. If the seller’s price is equal or lower to the regulator’s price, the patient will get access to the new therapy, resulting in increased life expectancy (months m) and quality of life (percent q). The treatment will be paid by the payers (deducted from their accrued premiums) and the investors will be compensated with the reimbursed price (divided by two). The patient’s therapy outcome converts into an economic benefit since quality of life equals work ability in percent of a fulltime salary for a healthy person of 10,000$ per month or 120,000$ per year. Regulators and sellers receive the fixed salary for a healthy person. The pricing decision is limited to a price range between 50,000$ (representing the price of the current SoC) and 500,000$.

The circumstances of the reimbursement situation with its consequences on all seven stakeholders are expected to have an impact on players’ private reservation price decision, as well as on their decision for an effective price offer to their negotiation partner. To compare the two decision tasks, participants played in a first game the reservation price task in five rounds, followed by the price offer task for the same five rounds (game two). All relevant information about consequences described above were introduced and trained before the first game. To prevent strategic behaviour, participants did neither know which round or game would be relevant for final payoffs, nor that the game would be repeated after five rounds with a slight adjustment of the task. In other words, the reservation price was not reported before but rather as a part of the negotiation. Of course, learning effects and assumptions about a “strategically optimal” reservation price might lead to bias. We tested in our previous study in a separate run of the experiment with another population (*n* = 201) differences between the two consecutive reservation games (with a role switch in-between, instead of an offer game). Further, we compared these two reservation price games with the first game of the population analysed here. No significant differences were found between mean reservation prices per rounds for our sub-population in focus [15].

Based on these findings we would expect participants to state in game two a price offer that equals the respective reservation price of the same round in game one since stated social preferences reflect all relevant payoffs, including the impact of a successful or forgone agreement. A rational decider would have no incentive to deviate if payoff-functions in both games were equal. However, to simulate a setting closer to a real-life situation, we adjusted the incentive structure for game two as displayed in Table 2. Deciders’ payoff-functions were extended to incentivize an agency relationship with one of the funders each. Sellers could keep the positive difference between their reservation price and their offer (margin) if their offer lead to a successful agreement as bonus. Similarly, regulators were rewarded with the positive difference between their submitted reservation price and any price offer below if an agreement was reached.

**Table 2:** Design of experiment (parameters per role and round)

State	Round	Reser-vation price^1^	Price offer^1,2^	Deciders				Receiver			Funders	
				Regulator		Seller		Patient			2 Payers	2 Investors
				Benefit^1,2^		Benefit^1,2^		Survival (m)	Quality of Life (q)	Benefit^1^	Benefit^1,3^	Benefit^1,3^
				fix	bonus	fix	bonus					
State without product								0	50%	0		
Initial state (SoC)	0	50	50	120	x-y	120	y-x	5	50%	25	240 - y	y
New product	1	x	y	120	x-y	120	y-x	8	50%	40	240 - y	y
	2	x	y	120	x-y	120	y-x	10	50%	50	240 - y	y
	3	x	y	120	x-y	120	y-x	12	50%	60	240 - y	y
	4	x	y	120	x-y	120	y-x	15	50%	75	240 - y	y
	5	x	y	120	x-y	120	y-x	17	50%	85	240 - y	y

1: for groups 3 and 4, amounts in ,000 $ (converted 100,000 $ = 1 US$ at the end of the experiment); for groups 5 and 6, amounts divided by 100 and displayed as $ (converted 1 $ = 1 US$ at the end of the experiment)

2: additional bonus for successful offer in second game (if agreement possible, y_S_ ≤ y_R_)

3. in the first game players see resulting benefits for funders, based on the potential reservation price (240-x and x)

SoC, standard of care (status quo); m, survival in months; q, quality of life on a scale of 1–100%

As described and trained in the introduction of the experiment, a successful agreement was assumed with a seller’s price below or equal to the regulator’s price (y_S_ ≤ y_R_). Successful agreements were determined by a randomized matching per round after the experiment. This instruction to the bonus mechanism (see Additional file 1) was the only supplementary information provided for the second game compared to the first one. The modification of the task during the experiment was not announced in the introduction.

### Research questions and hypotheses

Our underlying model builds on the “robust finding” from existing laboratory research in economics that “individuals take into account the welfare of all parties and have a preference for efficient outcomes” and that “non-selfish preferences are the rule rather than the exception” [15, 37–42]. Consequently we expect a rational regulator or seller to maximize his or her social utility considering own payoffs, as well as the utility functions of the other involved stakeholders. Of course, they can weight each utility differently, also with zero. The extended utility function makes participants adjust their social optimization task. They could tend to maximize own (selfish), overall (prosocial) or even others’ (altruistic) benefit. As soon as they care for their own bonus, they face a trade-off between bonus amount and probability of an agreement. In this case, they should base their offer decision on an assumption about their counterpart’s offer (for more details about the model, see Additional file 2):

- Research question 1: Do participants deviate with their offer statement from their reservation price?

Players could assume that no valuation differences exist between roles with WTP=WTA for the same round. Based on this assumption, they should offer their reservation price since this maximizes expected overall social payoff, even though their own bonus is zero. They also have no reason to strive for a bonus if they assume a systematic valuation gap (WTP < WTA) since a successful offer would require them to offer a price above their WTP or below WTA, which violates the introduced and trained definition of a reservation price. Finally, this strategy is also dominant if they assume WTP > WTA, but care for the patient only, since this offer maximizes the chance of an agreement and increases the patient’s benefit compared to the status without agreement.

*Hypothesis H0-I: deciders will claim no margins and state a price offer (y) equal to their reservation price (x).*

- Research question 2: Do participants differ in their bargaining behaviour (margin claimed) if the price magnitude differs from the expected payoff magnitude, imitating “real world” prices of new oncology treatments?

Since prices convert into equal final payoffs, relative magnitude of the pricing decision should have no influence on the pricing decision of a rational decider, caring for the real social payoffs at the end of the experiment only.

*Hypothesis H0-II: margins claimed converted to payoff-magnitude do not differ between price groups for any round.*

- Research question 3: Do participants differ in their bargaining behaviour (margin claimed) depending on their role?

Players could have a more general assumption about the distribution of the counteroffer than described above. For example, they could assume that their counterpart’s proposal is more likely to be located at the mean of an expected price range of possible or realistic price offers. Assuming further that participants share a certain range of expected prices, rational deciders should differ in their offer decision, depending on their role. If two opposite players share the same reservation price below the expected counteroffer, only the seller should ask for a margin. Vice versa, if they have an equal preference above the expected mean only the regulator should place an offer below his WTP while the seller should offer his reservation price [for a more detailed derivation, see Additional file 2]. However, a less rational player, interested in realizing a bonus, might just stick to a simple fix rule (e.g. “one percent margin”). In this case, margins between roles should not differ.

*Hypothesis H0-III: margins claimed do not differ between role groups for any round.*

- Research question 4: Does the result of the negotiation differ between rounds and price groups? What are the consequences for all involved stakeholders?

Not all dominant strategies described above result in an optimal social outcome from the decider’s perspective, even if we assume no valuation gaps and players to be rational utility maximizers. Nonetheless, if we assume that they prefer an agreement to none, we would expect; the more valid their assumption about each other’s price offers, the more successful agreements.

*Hypothesis H0-IV: Comparing price offers between the two role groups, an agreement is reached with means*${\overline{y}}_S$*≤*${\overline{y}}_R$*overall (weak) and for each negotiation pair y*_*Si*_ *≤ y*_*Ri*_*(strong).*

### Implementation

We recruited US residents aged 18 years or older. Participants had to state an informed consent prior to the experiment. Only MTurk users who did not participate in our previous run of the experiment (reported in our previous study [15]) were allowed to participate. No further restrictions for participation were defined. Instead we surveyed additional demographic information, as well as risk behaviour and health experience of each participant (see Appendix 4 of our previous study [15]). The variables were used to control the results reported, combined with the data from two attention screening / comprehension control questions [15, 35, 43]. Before the first game, both roles were introduced to and trained on the concept of a reservation price, the negotiation setting as well as on the consequences of their decisions. WTP was defined as the “absolute maximum price” regulators would “still consider reasonable and fair for the new product”, WTA as the “absolute minimum price” still considered “reasonable and fair for the new product” by sellers. Details on the design in general and the reservation price game specifically are laid out in our previous study [15]. Since a consistent price offer in the second game had to be equal or higher than the reservation price for sellers and vice versa for regulators, participants were provided with a message if their offer contradicted this requirement (Additional file 1). However, participants were allowed to ignore the message and submit inconsistent offers.

The design aims at understanding WTP-WTA differences in an interactive reimbursement setting. This implied a sufficiently high number of comparable decision situations for the matching at the end as well as several consecutive rounds (with increasing survival) at a reasonable duration of the experiment. We applied a contingent valuation method instead of a dichotomous choice format, which is the preferred option in experiments interested in nominal QALY preference statements [15, 44]. Due to the high complexity of the decision task, combining preferences for QALYs with social preferences, we refrained from the application of a Becker-DeGroot-Marschak (BDM) mechanism, which is in line with comparable experiments on social preferences in health care [15, 45–51].

### Statistical methods

We performed Chi-square, Cramer’s V, Fisher’s exact and Spearman rho tests, independent and paired samples t-tests, as well as independent Mann-Whitney-U-tests.

## Results

For the following, prices in the 100 k$ group are converted to the payoff magnitude for comparison with the 1$ group. Further, we focus on individuals with strict monotone preferences for incremental patient benefit; see also our previous study for a discussion of non-monotone preferences [15]. The offer game of this study expands our set of performance variables (preference structure, comprehension question regarding patient benefit, screening question to capture attentive players) with a fourth one: offer consistency. Participants were introduced and trained to the appropriate relation of reservation price and price offer. Their preferences submitted in the first game were further displayed in every decision of the second game below the decision table. If they moved the slider to an inconsistent offer, a message was displayed (Additional file 1). 90% of players overall (including those with non-monotone preferences) submitted at least one consistent offer. 64% of players with strict monotone preferences placed only consistent offers, compared to 27% in the non-monotone group. We will control for offer consistency in the following as indicated.

### Reservation prices considering offer consistency

We can confirm the results from our previous study if we control the reservation prices submitted with the additional information of consistent offer behaviour. Regulators’ reservation prices are still lower in the 100$k group compared to the 1$ group (t-test and U-test *p* < 0.05). We can likewise confirm the absence of valuations gaps looking at consistent players only. The negative gap (“preference range”) between roles found in the 1$ group can be confirmed as well (three rounds with t-test *p* < 0.05 for players with consistent offers in all five rounds, four rounds with t-test and two rounds with U-test *p* < 0.05 if we exclude inconsistent offers for each round separately).

### Bargaining behaviour: price offers

In line with the increasing reservation prices, price offers increased throughout the game from round one to five (t-test *p* < 0.01). Consistent offers were for all four groups and five rounds below the reservation price for regulators (H0-I rejected with t-test at *p* < 0.5 for two pairs, all others *p* < 0.01), respectively above the reservation price for sellers (*p* < 0.01).

**Fig. 1:**
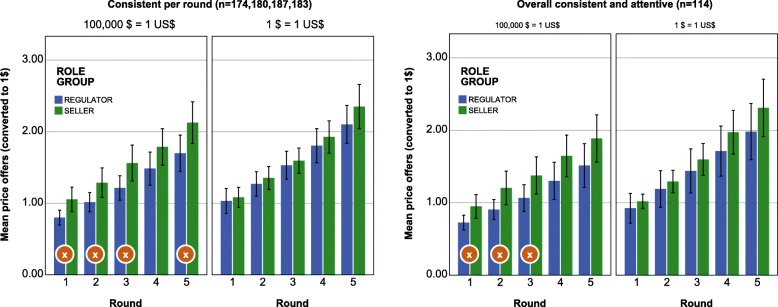
Price offers in the second game, split by price group and role. Confidence intervals: 95%. Rounds marked with “x”: mean price offer significantly different between roles at *p* < 0.05, hence no trade possible. Left side: inconsistent price offers (<WTA or  > WTP) excluded for each round separately. Right side: only participants with consistent offers in all rounds (players with one or more inconsistent offer excluded from all rounds) and correct answer to attention screening question

As displayed in Fig. 1, price offers of sellers were significantly higher in four of five rounds in the 100 k$ group, compared to those of the regulators (t-test *p* < 0.05). In consequence, an agreement between the average negotiators in this price group was only possible in one round (weak H0-IV rejected). For participants with consistent offers in all rounds and correct answer to the attention screening question, the effect holds for the first three rounds (t-test *p* < 0.05, Mann-Whitney *p* < 0.01). Differences between negotiators in the 1$ group were not significant (t-test and Mann-Whitney *p* > 0.05, except first round Mann-Whitney *p* < 0.01). The negative valuation gap found in the latter group might be an explanation for this result, but we have to look at the margins in both groups to see bargaining differences between the two price framings.

### Bargaining behaviour: margins

Margins claimed by participants increased throughout the game from round one to five (t-test *p* < 0.01 for all round-pairs, except last rounds for overall consistent players *p* < 0.05 respectively insignificant with consistency filtered per round). The increase is also significant for the majority of rounds if controlled for role or price group separately and for half of the rounds if controlled for treatment groups (t-tests *p* < 0.05). Margins between price groups differed only in one round per role group (H0-II rejected at *p* < 0.5 for the first round in sellers with U-test and the second round in regulators with t-test and U-test). Sellers in the 1$ price group claimed in the first round a higher margin than regulators (H0-III rejected at *p* < 0.5 with U-test). Margins between roles in the 100 k$ did not differ in any round. Hence, H0-II and H0-III cannot be rejected for the majority of rounds.

We found no evidence that players shared any general assumption about their opponent’s most likely offer, resulting in a break point where they would reduce their margin to zero. We tested fix break points for reservation price - margin pairs overall at 1.2$ (equality for funders benefit), 2.5$ (mean of price range) and 2.75$ (mean of slider range), as well as relative break points at reservation price quartiles. All role and treatment groups had above or below any tested break point mean margins different from zero (t-tests *p* < 0.05).

However, only 53% of players submitted in all five rounds a price offer that was different from their reservation price, while 47% claimed at least once no margin. 15% claimed no margin in all five rounds. Based on the assumption of rational utility maximization this could indicate that those players either assumed WTP=WTA or expected a valuation gap (WTP < WTA). It could also indicate that they only cared for the increased patient benefit and therefore chose the best offer to reach an agreement, and hence neglected any other payoffs, including the probability of a bonus for themselves.

### Negotiation outcome: agreements possible

As described above, mean negotiators in the 100 k$ did not reach an agreement in the majority of rounds, while in the 1$ group an agreement was possible on average. As alternative to comparing mean negotiators, we can match regulator and sellers on an individual basis.

We paired consistent offers per round randomly (a method applied before e.g. by Borges et al. [52]), split by price group. This was repeated for 500 iterations (states of the society) for the five rounds (products) and the two price groups (framings) for a sample of 5000 market states, each representing an average outcome of all its randomly matched offer pairs. Regulators and sellers with monotone preferences and consistent offers were matched to 45 pairs on average. Table 3 provides an overview of the main outcomes of the average state of the ten markets. Sixty one percent of the negotiation couples could have reached an agreement (“trades possible”), since WTP ≥ WTA. Yet, only 63% of these were successful, leaving on average 61% of patients with no access to the new treatment.

**Table 3:** Random pairing of consistent offers based on monotone preferences – mean of market outcomes

Round	Price group	Pairs	Trades possible based on monotone preferences				Successful trades based on consistent offers			Bonuses realized due to successful negotiation									
			number of trades		in percent of pairs		number of trades		in percent of pairs	regulators with bonus		average bonus in $ (for >0)		sellers with bonus		average bonus in $ (for >0)		total average bonus in $ (for >0)	
1	100k$	41	22.0		53.6%		12.6		30.7%	6.8		0.22		8.2		0.09		0.15	
	1$	43	29.9		69.5%	b1	17.3		40.2%	10.6		0.17		12.7		0.17	b2	0.17	
2	100k$	42	24.0		57.0%		14.7	b3	35.1%	7.9		0.41		9.5		0.12		0.25	
	1$	46	32.2		69.9%	b1,b4	20.0		43.5%	14.8		0.22	a1	15.5		0.17	b2	0.19	
3	100k$	45	24.7	b5	54.8%		14.7	b3	32.6%	9.8		0.33		10.9	b6	0.15		0.23	
	1$	48	33.6		70.1%	b4	21.4		44.7%	15.6		0.23	a1	16.4	a2	0.20	a3	0.21	
4	100k$	43	24.5	b5	57.0%		15.6	b7	36.3%	10.7	b8	0.36	b9	10.9	b6,b10	0.17		0.26	
	1$	46	30.7		66.8%		20.2	b11	43.9%	15.0		0.33		16.7	a2	0.20	a3	0.26	a4
5	100k$	44	23.3		52.9%		15.5	b7	35.2%	10.7	b8	0.36	b9	11.0	b10	0.19		0.27	
	1$	47	29.8		63.3%		20.3	b11	43.1%	16.1		0.29		16.3		0.24		0.27	a4
**average**		**45**	**27**		**61%**		**17**		**39%**	***63% of trades possible***									

500 iterations (states of the society) for 10 markets (5 products in 2 price frames). Iterations are not correlated with the outcomes. Cases analysed are 5000 market states, not participants. There were no double entries to remove; each market state was unique

Consistent price offers (<WTA or > WTP) excluded for each round separately, instead of excluding inconsistent players (one or more inconsistent offers) overall

Rounds: Independent sample t-test between rounds (consecutive, controlled for price group) significantly different at *p* < 0.01 for all columns, except pairs marked with letter “a” (*p* < 0.05) and “b” (not significant)

Price groups: Independent sample t-test between price groups for all rounds significantly different at *p* < 0.01 for all columns, except total bonus realized in round 5 *p* < 0.05, in round 4 not significant

Trades successful: Paired sample t-test (one-tailed) for same iteration significant for differences between trades possible vs. successful trades (*p* < 0.01)

Roles: Paired sample t-test for same iteration significant for differences between roles regarding number of players with positive bonus (*p* < 0.01, except for 100 k$ round 4 *p* < 0.05; not significant for 1$ round 5) and average bonus for players with bonus (*p* < 0.01, except not significant for 1$ round 1)

The number of unique combinations in each market is high (e.g. for round 1 of 100 k$: 43!/(43–41)!). We increased the number of iterations up to a point where the variables of interest were not significantly different anymore between two runs with the same iteration size and the difference of average trades possible and trades successful (in percent of pairs) between the runs were stable at 0.1%

**Fig. 2:**
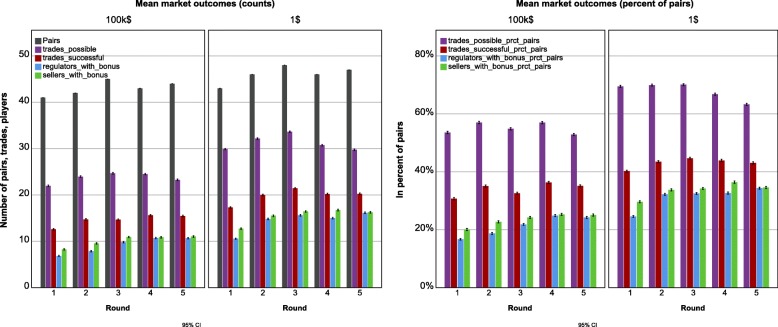
Mean of market outcomes. Limitation: The 500 markets simulate the societal effect of the fixed preferences and offers of on average 91 individuals randomly paired. Differences in a sample of 500 unique pairs of the investigated population might be less clear due to higher variance. However, the relevant differences in the simulation were based on statistically significant differences in the source population, as shown. CI, confidence intervals

Negotiation pairs increased for both price groups compared to round one with a maximum in round three, which is directly related to the number of consistent price offers per round. Controlling for this, the percentage of trades possible was still significantly different between consecutive rounds for the 100 k$ group and between round three to five for the 1$ group (*p* < 0.01). Further, the percentage of successful trades differed between all consecutive rounds in both price groups, as well as between price groups in all rounds (*p* < 0.01). As displayed in Fig. 2, the percentage of trades possible declined over the five rounds (*p* < 0.01 in the 1$, *p* < 0.05 in the 100 k$ group), while the percentage of successful trades increased (*p* < 0.01). However, at least the 1$ group had a peak in round three with declining agreements after and in the 100 k$ group the increase was inconsistent with two increases and two declines (*p* < 0.01). Given the trades possible, the percentage of realized trades increased between the first and last round for both price groups (*p* < 0.01). This effect was paralleled by a clear increase in average bonus for successful negotiators, as well as for the average player overall, since the number of players with bonus increased for both roles (*p* < 0.01).

**Fig. 3:**
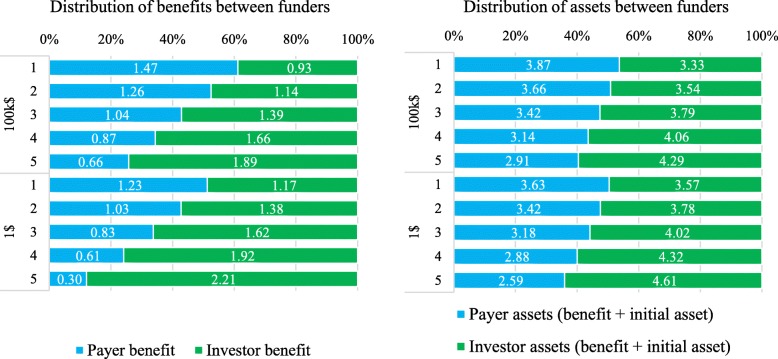
Mean of market outcomes – funders’ benefits and payoffs from round 1 to 5 per price group (means for successful pairs). Total benefit for redistribution by players in all rounds equals 2.4$ (1.2$ premiums from each payer). Total assets equal of funders equal benefit of 2.4$ (premiums unused for payers and price income for investors) plus initial assets of 4.8$ (1.2$ for each of the payers and investors). Total assets were divided by ten for final payoff after experiment

All market outcomes displayed in Table 3 were significantly different between the two price groups in all rounds (except total bonus in round 4). The 100 k$ group was significantly and notably less successful in negotiating agreements than the 1$ group. This holds not only after controlling for the number of negotiation pairs (*p* < 0.01), but also if we compare successful trades relative to trades possible for both groups (round three to five at *p* < 0.01). However, the 1$ group closed negotiations also at 19% higher market prices on average. In consequence, the redistribution of constant assets between funders over the five innovation cycles was different between the two framings (see Fig. 3). While successful negotiators in the1$ group started with an even distribution, they allocated in the last round 36% to the payers versus 64% for the investors. The 100 k$ group allocated in all rounds significantly less to the investors respectively more to the payers, starting at 54 to 46%, leaving them a share of 40 to 60% in the final round.

## Discussion

There is a high need to understand how existing pricing policies for new pharmaceuticals are effective in reaching conflicting societal goals, like timely access, sustainable health budgets, or incentivizing research in “value for money” rather than “me-too” therapies [28]. However, not only the pricing mechanics, also the process of value assessment and price negotiation is expected to have an impact [1, 14]. In particular because also the implementation of value-based pricing does not imply that negotiation ranges disappear [1]. While these ranges may arise from information asymmetries (research and development costs, clinical benefit) [1], they might as well arise from behavioural factors [14]. The paper presented here concludes our test of an experimental design [15] to analyse pricing negotiations for pharmaceuticals from a behavioural perspective. Available information as well as payoff functions were equal between the two negotiating roles (apart from the bonus direction in the bonus game). Based on our model, we would have expected rational players to reveal no valuation gaps, no claimed margins and achieve agreements throughout the game. The results of this study show that even if mean negotiators would agree on a “fair and reasonable” price (no valuation gaps), a majority of reimbursement negotiations can still fail because WTP and WTA between regulators and sellers may differ on the level of individual negotiations. Societal consequences differ, related to the price magnitude, with relatively lower patient benefits but also lower payer costs at current price levels of new oncology treatments. At least two possible target points for the current policy reform debate can be derived from the results: price related framing effects, as well as role related behavioural effects.

The framing effect of the price magnitude should be investigated further as potential policy intervention target to affect prices for and access to new pharmaceutical treatments. Relatively lower prices for high cost therapies could reflect a concern regarding budget impact. It could just as well mean, that negotiators tend to neglect the same at lower price levels. Causing higher budget impact for large treatment populations at lower price (per treatment) levels. Moreover, if negotiators incorporate budget impact considerations, this should preferably not decrease the number of agreements and access for patients. Otherwise patients suffering from a high-cost disease would be discriminated compared other patient groups.

From a practical behavioural policy perspective, transparent official reporting on access differences between treatment areas might be a relatively easy real world intervention. In analogy to governmental information campaigning as policy instrument for behaviour change (“sermon”) in other policy areas [53]. In addition, further experimental research could investigate, whether price negotiations focusing on incremental cost-effectiveness, rather than nominal prices, align negotiation results between different price magnitudes – for better or worse, depending on the policy interest. Especially since cost-effectiveness has been found to be the most important predictor for reimbursement decisions in countries like the United Kingdom [13, 15, 54].

The primary interest of our design presented was to uncover role related differences affecting negotiation outcomes. The introduced bonus in the offer game caused players to departure from an optimal price, reducing the probability of an agreement. The prospect of a price-related reward might have triggered either (“negatively”) a loss-aversion in the sense of regret avoidance (“bad-deal aversion”) [55–57] or (“positively”) an overestimation of the bonus probability. The “coercive paternalist” approach [58, 59] would be to just prohibit the negotiators’ principals from introducing any price-related incentives for their agents to reduce the negative impact of bonuses on patient access. As an analogy: for providers in health care the potential negative impact of bonuses on patient outcome has been widely studied [60–62] eventually inspiring policy reforms to control them (e.g. just recently in Switzerland [63, 64]). Yet, to assume that potential offer gaps in real world are mainly driven by identifiable bonus payments, paid by the negotiators’ principals, would be an implausible simplification (especially since bonuses might play a role mainly on the seller side in many health systems). Experimental research in general has shown, that “people are concerned with unobservable payoffs such as reputation, fairness or the well-being of others” [65]. Anticipated regrets alone are likely to relate to much more than just a direct payment. They could for example be linked to general “valuation and trade uncertainties” [15, 66, 67]. Offer gaps in general might also arise from other behavioural effects like a “different focus” of the seller or buyer role [68–73], or role related moral commitments [74, 75], to name a few of many other proposed explanatory heuristics for valuation gaps, discovered in experimental studies [15, 22].

Again, public information could help by making the “hazardous behaviour” more transparent. Assuming of course, that an agreement is not only in the interest of both parties, but also economically viable and at least partly prevented or delayed by bounded-rational behaviour. In this regard the “Patients W.A.I.T.” indicator, published by the European Federation of Pharmaceutical Industries (EFPIA) [17] should at minimum benchmark patient access differences (availability and delays) for both sides. Not only between countries, but also between market authorization holders. If an official “patient waiting time” for reimbursement negotiations would be made transparent on an entity level (company, agency, etc.), the policy instrument would be even more promising, providing a strong “nudge” towards finding an agreement rather than “sermonizing” it in the name of the patient. For example in the form of a traffic light system indicating the “access quality” of negotiations, nudging not the consumer (see labels for food items [76]) but the responsible supplier. A red light status might be comparable to the stigma of standing exposed in the “only slightly less convenient” smokers area [77]. Or, without the shaming, comparable to the field experiment of Hallsworth et al. where general practitioners with a “prescribing rate for antibiotics” in the top 20% received a letter informing them “that the practice was prescribing antibiotics at a higher rate than 80%” of their peers [78]. While this “nudge” had a positive effect, the later sent out “patient-focused information that promoted reduced use antibiotic use” (sermon, after re-randomization) hat no effect on antibiotics dispensed [53, 78]. Promising behavioural policy interventions in the context of this study should promote the desired outcomes of reimbursement negotiations: more successful agreements (availability), reached in less negotiation time (time to access), at a cost-effective (value for money) and sustainable (affordability) price level (other outcomes see here [28]).

The starting point for policy reforms, using behavioural science, could indeed be to transfer and adapt behavioural tools from other policy areas. However, there is probably no way around developing specific behavioural tools to solve existing challenges in pharmaceutical pricing. Further experimental research should investigate interventions that shift price agreements closer to stated preferences and increase the number of trades realized towards the number of trades possible. This could stimulate reform efforts in European markets were price agreements are increasingly held confidential and thus can neither be analysed on an actual basis nor be compared to a publicly debated willingness to pay [1, 10, 11, 79, 80].

To judge the usefulness of an experimental approach to inform policy reforms we will briefly discuss at the end two potential limitations regarding external validity: the applicability of the experimental setting (decision situation) and the transferability of the observed behaviour (decision takers) in the experiment to a real world price negotiation. We will however not review the ongoing debate on validity of economic laboratory experiments in general [27, 81–87].

A central prerequisite for a credible applicability is the incentive compatibility of the decision situation. While our survey method was in line with comparable experiments on social preferences in health care [15, 45–50] we cannot rule out that stated preferences in the first games might be biased by certain strategic behaviour as discussed above. However, the optimal price in the offer game is not affected by any strategic behaviour in the first game (only the bonus probability is) and any strategic behaviour in the second game was exactly at the centre of our interest. The experimental payoffs were in line with comparable experiments as well [15, 45–50]. The indirect representation of the patient as receiver by a donation to a cancer patient supporting foundation reduces undeniably the applicability of the design. An implementable and ethically justifiable alternative is to our knowledge not available. The applicability of the setting could be further increased, based on existing experimental evidence. Goal should be to increase its capability to measure generalizable outcome differences, sensitive to relevant (effective, implementable) interventions. External validity of laboratory experiments in general, and social-preference games in particular might depend to a great extent on a relevant context and avoidance of artificiality [81, 82, 84, 86]. Arlen and Tontrup for example showed in a series of experiments that negotiators “can mute regret by trading through institutions that let them share responsibility with others” [57]. Since negotiators in real world reimbursement negotiations, on the selling and the buying side, usually have to go through approval processes, a shared decision situation in the design could be a promising option to further increase applicability. Another promising and very relevant adjustment to the design could be to vary initial price anchors (different standard of care comparators, or opening offers from the buyer or seller). To understand its potential impact on agreed prices overall [14], but also its potential impact on the share of successful agreements.

The general transferability of the observed behaviour from laboratory participants to actors in the real world is equally important as the applicability of the setting. Main concern is the relevance of specific knowledge and experience for the task, which is expected to differ between mean recruited participants in the experiment versus professional negotiators in the setting of interest.

The impact of trading experience and sophistication on behavioural biases has been studied using data from the laboratory as well as field data from financial market trades, as well as combined data [22, 88–99]. Empirical studies using real market data generally show that experience reduces behavioural biases, but biases remain relevant even for experienced traders [65, 93–100].. Specifically the disposition effect (tendency to sell wining positions too early and holding losing positions too long), linked to regret avoidance, is present in experienced traders as well [93–97, 99–101]. The evidence from the laboratory is largely based on a series of experiments from List et al. who found that experience in trading simple objects reduces reluctance to trade [22, 88–91]. The setting compares the behaviour of untrained versus experienced field traders in trading “pins, sports cards and sports memorabilia” [90]. While the linking of laboratory and field is very interesting, it is unfortunately only in very few, specific markets possible. Further, the simple good transactions analysed lack some very decisive characteristics compared to other markets, which are known to trigger behavioural effects. To name just three, relevant in our case:

i) Higher and changing complexity of product characteristics: Evidence from various experiments has revealed that sellers and buyers in fact do “tend to focus on different aspects of traded goods”, which causes exchange gaps [22, 68–73]. This effect is expected to be higher in our market, due to the complexity of actual pharmaceutical therapies and target indications. The characteristics in a real trade situation for sports cards or pins on the other side are likely to be much more comparable and stable, between lab and field, and much more repetitive over recurring negotiations.
ii) Higher uncertainty of product value and trade outcome: Engelmann et al. who replicate and adapt the results of List et al. identify “trade uncertainty” as the relevant driver for exchange asymmetries “which may well be perceived in markets” outside the laboratory [66]. As they argue, “markets may not provide sufficient incentives to explore new strategies that help to overcome exchange asymmetry, hence the asymmetry persists” [66].. According to our opinion (and the first authors own experience from respective reimbursement negotiations on both sides) the subjects of the negotiation as well as the relevant rules of the game itself are often much less constant between each “reimbursement game” than in other markets. Even for a specialized company or regulating agency two consecutive products under negotiation (even in the same therapeutic area) can differ decisively regarding body of evidence (value), relevant comparators (alternative) and price magnitude. New forms of managed entry agreements add even more complexity to this [1, 102, 103]. In parallel, the constant reform efforts in most European countries [5, 104], combined with the growing professionalization of regulating agencies, including internal regulating bodies in the pharmaceutical companies [105], change the rules of the game on a recurring basis.
iii) Irreversibility (lack of tradability). Various experiments have contributed to the better understanding of regret avoidance and “bad-deal aversion” [22, 55–57, 67, 106, 107]. Ratan found in an experiment, where one group of participants could reverse their decision during 24 h, while for the others it was final, no valuation gaps in the group with the option to reconsider, while the other group revealed reluctance to trade [67]. Normally price agreements reached for official reimbursement status are finite and cannot be reviewed for a certain time (or at least not without considerable legal efforts). Both sides face the threat that new, relevant information becomes available after the decision, which could make the quality of the decision and the negotiation success questionable. This might again be different of course in a lot of other markets for simple commodities where prices can be adjusted without governmental approval and “bad deals” bought can be resold.
iv) Moral valence: Some experiments reported results that suggest, that trades triggering “moral responsibility”, as well as public goods, induce higher valuation gaps [22, 74, 75, 108, 109]. In the study presented here regulators and sellers reported a different relevance of involved stakeholders for their decision (“regulators rated the patient’s relevance for their decision higher and the own role lower compared to sellers”, see previous study) [15]. Experienced professionals deal on a daily basis with their responsibility to attach “a price-tag” to life-saving treatments. It is possible, that they refrain much more or fully from a potential morally induced valuation gap. On the other hand, it could just as well be that the different professional environments for buyers and sellers, with selected information provided by differing interests of each principal, lead to different opinions that drive price expectations apart.

Finally it is important to emphasise, that results from laboratory experiments have to be complemented and bridged with non-experimental methods [27, 110]. Compared to the lab-field comparisons in behavioural finance it might to date be inherently more difficult to collect reliable quantitative data on pharmaceutical price negotiations outside the laboratory. There is not only a lack of data on the course of negotiations (e.g. duration from first offer to agreement, number of offers exchanged, etc.) but also of the prices actually negotiated, since these are in most European countries not publicly available [1, 10, 11, 79, 80]. Against this background, observational data collected via expert surveys can serve as promising complement (see Vogler 2018 who provides a comprehensive review and guidance on price surveys in general, including surveys with competent authorities [111], as well as [11, 79, 80].

## Conclusions

The incremental value of new pharmaceuticals as well as the willingness to pay for an additional, quality-adjusted life year (QALY) are central in current policy reform debates [1, 5–10, 15]. There is a clear need to comprehend the capacity of “value-based” pricing policies to impact societal goals, like timely access, sustainable health budgets, or incentivizing research to improve patient outcomes [15]. However, not only the pricing methods but also the reimbursement and pricing processes are subject to reform demands in this regard [1, 11–15]. The study presented here aims to enrich the debate from a behavioural perspective. Since “behavioural economic-related phenomena may affect price negotiations” for new pharmaceuticals as they affect professional deciders in other markets [65, 93–100]. The findings show that even if average regulators and sellers could agree based on their stated preferences, a majority of reimbursement negotiations can still fail due to valuation gaps in individual negotiation situations. The societal consequences differ, related to the price magnitude of the therapies. The price level of current new oncology treatments leads to lower patient access, but also lower payer costs, compared to a treatment in a low-price frame.

From a practical behavioural policy perspective, transparent official reporting on negotiation outcome differences on an entity and treatment area level might serve as a nudging policy to address potential behavioural flaws. In addition to transferring behavioural tools from other policy areas, further experimental research should investigate how specific interventions can improve reimbursement negotiation outcomes for new pharmaceuticals. Promising behavioural policy interventions should promote socially desirable outcomes, such as availability, time to access, value for money, affordability and equity.

## Supplementary information

**Additional file 1.** Selected screens and payoff details.

**Additional file 2.** Model and hypotheses.

## Data Availability

Experimental instructions and datasets (fully anonymized) analysed during the current study are available in the zenodo repository, 10.5281/zenodo.3575971 upon request.

## Abbreviations

100 k$: 100,000 $
H0: Null hypothesis
ICER: Incremental cost-effectiveness ratio
MTurk: Amazon Mechanical Turk
OECD: Organisation for Economic Co-operation and Development
QALY: Quality-adjusted life year
SoC: Standard of care
USD: US Dollar
WTA: Willingness to accept
WTP: Willingness to pay
