# Supplementary material for: The impact of reimbursement negotiations on cost and availability of new pharmaceuticals: evidence from an online experiment
Source: Health Econ Rev. 2020 May 21;10:13. doi: 10.1186/s13561-020-00267-y (PMC7243324; doi:10.1186/s13561-020-00267-y)
Supplement: Supplementary file 1 — Additional file 1. Selected screens and payoff details. [file 13561_2020_267_MOESM1_ESM.pdf]

Appendix 1: Selected screens and payoff details

Figure 4 and 5: introduction to price offer task (left for the regulator, right for the seller role)

We will revisit the five new pharmaceutical products of round 1 to 5. But now you can state an actual price expectation to the seller. The seller himself will do the same. Your counterpart is another participant, randomly selected after the experiment.

If your expectation is **higher or equal** to the price offer of the seller, you will both agree. In consequence, the patient will get access to the new treatment with all its benefits (increased life expectancy, economic benefit). The investors will receive the price (revenue) and the payers will have to pay the price (cost). If no agreement is reached, the product will not be available in this country.

You and your counterpart will both receive **an additional bonus for reaching an agreement: you can keep the difference between** your successful price statement and your absolute maximum price (stated in the first part of the experiment). Hence, the lower your price statement compared to your absolute maximum price, the higher your bonus – if the offer is not below the seller's price statement. Then again, the higher your price expectation, the higher potentially the chance of reaching an agreement with the seller.

Below the decision table you will see for each round your stated maximum prices from the first part of the experiment.

Ok? Let us start!

We will revisit the five new pharmaceutical products of round 1 to 5. But now you can place an actual price offer to the regulator. The regulator himself will do the same and state his price expectation. Your counterpart is another participant, randomly selected after the experiment.

If your offer is **lower or equal** to the price expectation of the regulator, you will both agree. In consequence, the patient will get access to the new treatment with all its benefits (increased life expectancy, economic benefit). The investors will receive the price (revenue) and the payers will have to pay the price (cost). If no agreement is reached, the product will not be available in this country.

You and your counterpart will both receive **an additional bonus for reaching an agreement: you can keep the difference** between your successful price offer and your absolute minimum price (stated in the first part of the experiment). Hence, the higher your price offer compared to your absolute minimum price, the higher your bonus – if the offer is not beyond the regulator's price statement. Then again, the lower your price offer, the higher potentially the chance of reaching an agreement with the regulator.

Below the decision table you will see for each round your stated minimum prices from the first part of the experiment.

Ok? Let us start!

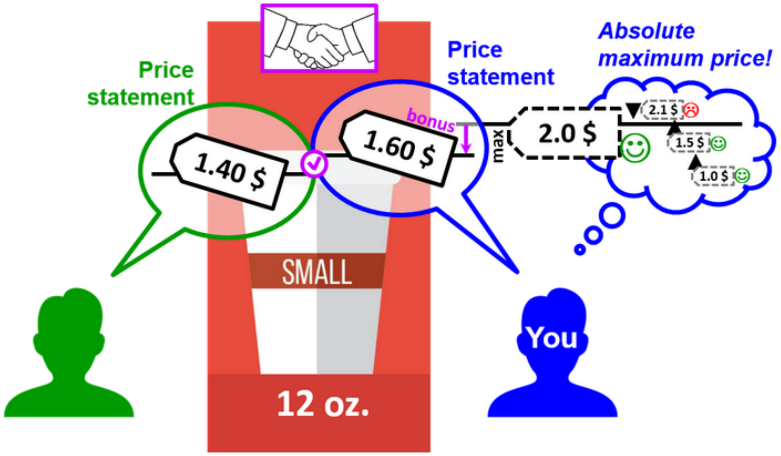

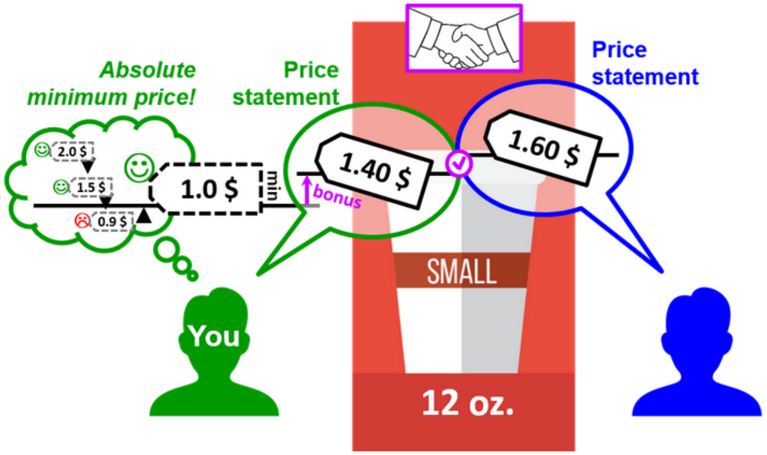

The coffee cup relates to the comprehension questions in the introductory training, see Appendix 1 in our previous publication [1]. Coffee cup adjusted from wikiHow [2].

Figure 6: decision table with message for price offers violating reservation price as introduced and trained (run two, example for seller role). Participants could still ignore the message and submit any price in range.

Your maximum price of 160000 $ leads to the following state of the society:			
in thousand Dollars	**New Benefit**	**Compared to Current State**	**New Asset (Benefit + Initial Asset)**
**Patient**	50	+25	50
**2 Payers**	80	-110	320
**2 Investors**	160	+110	400
**Seller**	120	0	240
**Regulator**	120	0	240

Your price statement is equal or below your minimum price. This might increase the chance of an agreement but you will not receive any bonus.

Please state your price by moving the red slider below.
You will see the related consequences in the table above.
If the values do not change, please click the red slider again.

Your minimum prices form the first five rounds:
8 months: 162,000 $
10 months: 160,000 $
12 months: 218,000 $
15 months: 348,000 $
17 months: 390,000 $

Payoffs to stakeholders:

The experiment was designed as Qualtrics survey and linked on MTurk as Human Intelligence Task (HIT). The Decision Science Laboratory of ETH Zurich (DeSciL) executed the experimental runs and delivered anonymized data to the researchers. Bonus distributions were performed by the DeSciL to ensure participants remain anonymous to the researchers.

- All prices expressed in fictive "Dollar" ($) during the game (group 1 and 2) traded at the end of the experiment at a currency rate of 100,000 $ = 1 US$. Prices for group 3 to 4 traded at par (see Table 1).
- Patient: benefit converted to US$ was donated to the Leukemia & Lymphoma Society (LLS) which provides financial support for patients with blood cancer (https://www.lls.org/support/financial-support).
- Payers and investors: benefit plus initial assets converted to US$ divided by ten was paid to four other MTurk-Users (randomly selected, only positive amounts implemented).
- Regulator or Seller: A fix amount of 2.4 US$ was paid to each participant (voucher right at the end of the survey) corresponding to a "yearly income" plus initial assets in the experiment. Bonus was distributed by the DeSciL, after the experiment was closed. Participants received the difference between their price offer and their reservation price, if their offer lead to an agreement for any successful round in game two. This was determined by a random pairing of regulators and sellers at the end of the experiment.

1. Wettstein DJ, Boes S. Assessing Social Preferences in Reimbursement Negotiations for New Pharmaceuticals: An Experimental Design to Analyse Willingness to Pay and Willingness to Accept *unpublished.* 2020.
2. Wikivisual. How to Order Coffee. wikihow.com: wikiHow Inc.; 2019.
