# Supplementary material for: The impact of reimbursement negotiations on cost and availability of new pharmaceuticals: evidence from an online experiment
Source: Health Econ Rev. 2020 May 21;10:13. doi: 10.1186/s13561-020-00267-y (PMC7243324; doi:10.1186/s13561-020-00267-y)
Supplement: Supplementary file 2 — Additional file 2. Model and hypotheses. [file 13561_2020_267_MOESM2_ESM.pdf]

Appendix 2: Model and hypotheses

Our underlying model is based on the "robust finding" from existing laboratory research in economics that "individuals take into account the welfare of all parties and have a preference for efficient outcomes" and that "non-selfish preferences are the rule rather than the exception" [1-7]. Relating to similar research, we built our reflections in the previous study on a simple CES[1]-function (see Appendix 2 of the publication) [7]: A rational decider (regulator R or seller S) should maximize his or her social utility (U) considering the utility (benefit π) of the other involved stakeholders besides his own with U ($\pi_R,\pi_S,\pi_C,\pi_P,\pi_I$), including the patient's (consumer C), the payers' (P) and the investors' (I) benefit. Payoffs in the reservation price games were for both deciding roles identical, independent of their decision (x) and flat during the game (fixed salary). Hence we proposed to simplify the utility functions for both deciding roles (D): U_D (π_C,π_P,π_I) = $(\gamma_D \pi_C^\rho + \delta_D \pi_P^\rho + \varepsilon_D \pi_I^\rho)^{1/\rho}$ with γ=1-δ-ε representing the distributed weighting of the affected passive stakeholders' benefits. [7]

For our reflections in the study presented here, we extend the utility function from above for the decider (D) with his and his opponents (O) benefit as follows:

U_D ($\pi_D,\pi_O,\pi_C,\pi_P,\pi_I$) = $(\alpha_D \pi_D^\rho + \beta_D \pi_O^\rho + \gamma_D \pi_C^\rho + \delta_D \pi_P^\rho + \varepsilon_D \pi_I^\rho)^{1/\rho} * Pr(y_D \,|E(y_O))$

with π_R^ρ = 240 + ($\hat{x}_R - y_R$) for regulators (D, O), π_S^ρ = 240 + ($y_S - \hat{x}_S$) for sellers (D, O), and α = 1-β-γ-δ-ε.

The estimated probability (Pr) for an agreement depends on the own price offer y_D in the second game and the expected price offer of the opponent y_O. The potential bonus depends on the own

[1] Constant elasticity of substitution

reservation price from the first game (external variable $\hat{x}_D$). A rational utility maximizing decider should maximize relevant payoffs considering the probability of reaching an agreement.

We first assume that no valuation differences exist, especially not between roles (WTP=WTA) and that players share this assumption with $x_D = x_O$ for the same round.

Hypothesis H0-I: deciders will state a price offer (y) equal to their reservation price (x), since this maximizes expected overall social payoff, even though their own bonus is zero.

We could further assume that rational deciders with social preferences might care for the patient only and weight all other payoffs with zero. A consequence of this assumption would be that the optimal price offer for the decider is again equal to his reservation price, but even if he assumes WTP≠WTA. Since this offer maximizes the chance of an agreement and increases the patient benefit compared to the status without agreement. Hence the result is equal to H0-I for altruistic players who do not assume WTP=WTA but care for patient's outcome only.

Alternatively we could assume that the deciders are still rational utility maximizers, but deviate from y=x since they do not only assume WTP≠WTA but do care for other stakeholders' benefits, beside the patient. For this, deciders need an alternative assumption for the opponents offer. If both roles simply assume a systematic valuation gap (reluctance to trade) where sellers value the new treatment higher than regulators (WTP < WTA) the outcome is again $y_D = x_D$ (H0-I). Since a successful offer with $y_S \leq x_R$ would violate the (introduced and trained) definition of WTP and WTA.

They could however have a general assumption about the distribution of the counteroffer $E(y_O)$, less dependent of their own valuation. For example that $E(y_O)$ is more likely to be located at the mean of an expected price range of possible or realistic price offers. A very simple expected price range is equal to the range between 0 and 500 k$ for which the decider could assume a normal

distribution of his opponent's offer y_O around 250 k$. Alternatively, the expected mean y_O could also be at 275 k$ (mean of the decision range). We further assume that participants share one range of expected prices in the experiment.

If the decider is rational and weights his own benefit higher than the benefit of his opponent, the optimum lies beyond y_O (>250 k$) for the regulator and below y_O for the seller (<250 k$).

> *Hypothesis H1-III: margins claimed do differ between role groups above or below a shared assumed most likely counteroffer.*

The smaller the decision maker estimates the standard deviation of the (normal) distribution of y_O, the steeper the density function and smaller the distance of his optimal expected bonus from the expected y_O (250 k$). This holds true also if the decider is strictly prosocial and weights all payoffs equal (see Figure 7 below). Only the optimum for an altruistic seller who weights the passive stakeholders equal but ignores not only his own but also the payoff for his active opponent, lies still at the point of equal distribution between funders at a price of 120 k$.

> Hypothesis H1-IIIa: Regulators will either state $y_R = E(y_O)$ if their reservation price from the first game is $\hat{x}_R \geq E(y_O)$, or $y_R = \hat{x}_R$ if their $\hat{x} < E(y_O)$. The sellers will state $y_S = E(y_O)$ if their $\hat{x}_S \leq E(y_O)$, or $y_R = \hat{x}_S$ if their reservation price is $\hat{x}_S > E(y_O)$.

This means for example for $E(y_O) = 250$ k$: regulators with a reservation price of 50 to 250 k$ as well as sellers with a reservation price of 250 to 500 k$ will not deviate with their offer from their reservation price. Since they would otherwise decrease the chance of an agreement and therefore expected payoffs. Regulators above 250 k$ however should deviate from their preference and ask for 250 k$, as well as sellers below 250 k$ and therefore have a "margin" between offer and reservation price.

Generalized H1-IIIa implies that if we split participants in two groups of those with reservation prices above and below the expected mean offer, regulators should have margins only in the upper and sellers only in the lower group.

Finally, we could in general assume that players share a valid assumption about each other's price offers.

- Hypothesis H0-IV: Comparing price offers between the two role groups, an agreement is reached with means $\overline{y}_S \leq \overline{y}_R$ overall (weak) and for each negotiation pair $y_{Si} \leq y_{Ri}$ (strong). Since players are rational utility maximizers and assuming that an agreement in line with their stated social preferences is always preferable to none

Figure 7: expected social utility for price offer decision in round 2 for each given reservation price, assuming that opponent's counter offer is normal distributed.

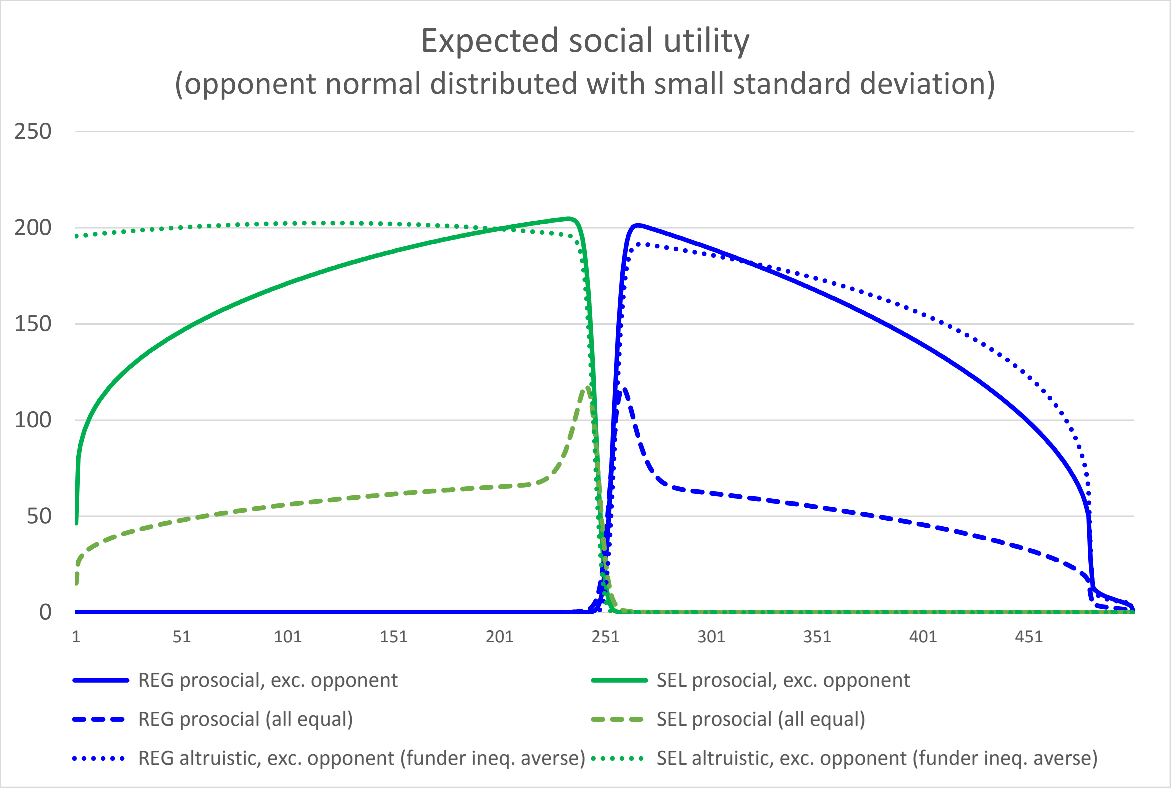

For simplification "no treatment" as default if no agreement is reached. Optima remain unchanged with "standard of care" as default. Fix salary of deciders excluded.

Figure 8: same if decider cares for patient/agreement only

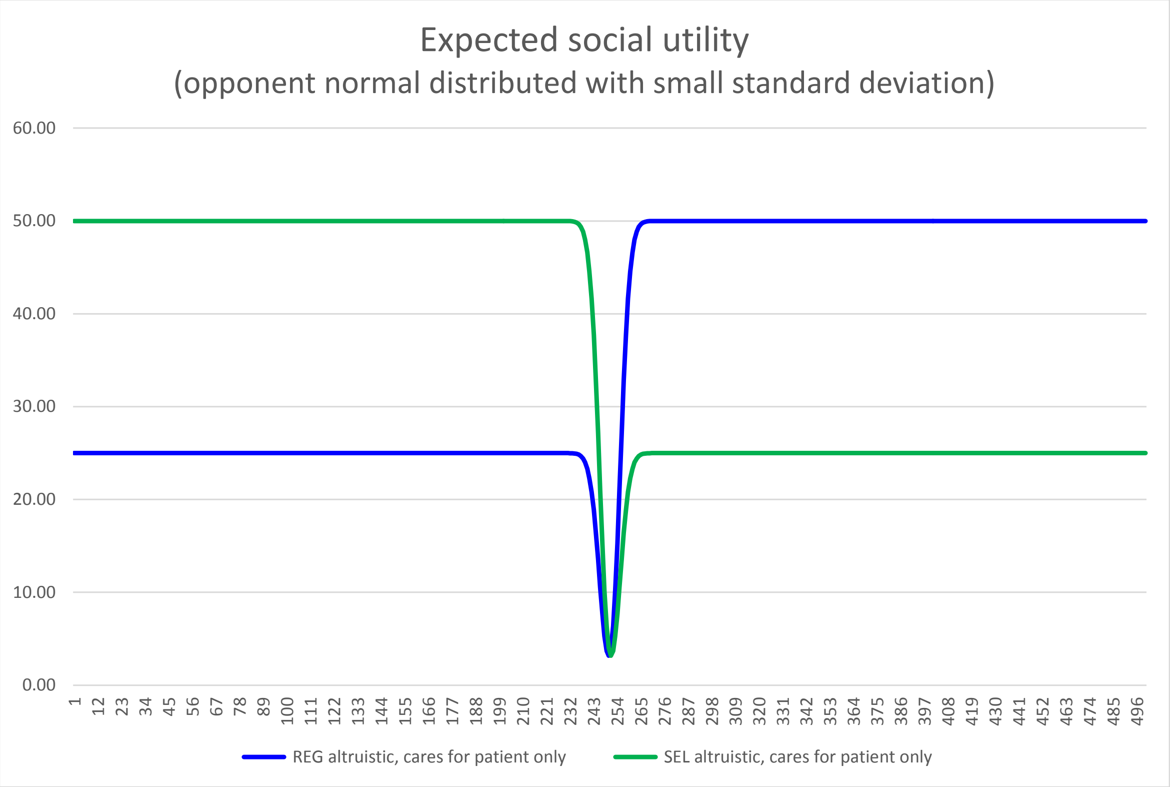

Standard of care as alternative, if no agreement is reached. Optima remain unchanged with "no treatment" as default.

1. Schumacher H, Kesternich I, Kosfeld M, Winter J. One, two, many—Insensitivity to group size in games with concentrated benefits and dispersed costs. *The Review of Economic Studies.* 2017;84(3):1346-1377.
2. Andreoni J, Miller J. Giving According to GARP: An Experimental Test of the Consistency of Preferences for Altruism. *Econometrica.* 2002;70(2):737-753.
3. Charness G, Rabin M. Understanding Social Preferences with Simple Tests. *The Quarterly Journal of Economics.* 2002;117(3):817-869.
4. Engelmann D, Strobel M. Inequality aversion, efficiency, and maximin preferences in simple distribution experiments. *American economic review.* 2004;94(4):857-869.
5. Fisman R, Kariv S, Markovits D. Individual preferences for giving. *American Economic Review.* 2007;97(5):1858-1876.
6. Bruhin A, Fehr E, Schunk D. The many faces of human sociality: Uncovering the distribution and stability of social preferences. *Journal of the European Economic Association.* 2018;17(4):1025-1069.
7. Wettstein DJ, Boes S. Assessing Social Preferences in Reimbursement Negotiations for New Pharmaceuticals: An Experimental Design to Analyse Willingness to Pay and Willingness to Accept *unpublished.* 2020.
